# Knowledge of direct obstetric causes of maternal mortality and associated factors among reproductive age women in Aneded woreda, Northwest Ethiopia; a cross-sectional study

**DOI:** 10.11604/pamj.2017.27.32.10274

**Published:** 2017-05-11

**Authors:** Fikreselassie Getachew, Getachew Mullu Kassa, Mulatu Ayana, Endawoke Amsalu

**Affiliations:** 1 Ethiopian Public Health Institute, Ethiopia; 2 College of Health Sciences, Debre Markos University, Debre Markos, Ethiopia

**Keywords:** Maternal mortality, knowledge, obstetric causes, women of reproductive age, Ethiopia

## Abstract

**Introduction:**

in Ethiopia, 20,000 women die each year from complications related to pregnancy and childbirth with much more maternal morbidity occurring for each maternal death. Good knowledge of women related with direct causes of maternal mortality is important in reducing maternal morbidity and mortality. Therefore, the aim of this study was to assess knowledge of direct obstetric causes of maternal mortality and associated factors among reproductive age of women in Aneded woreda, Northwest Ethiopia.

**Methods:**

A community-based cross-sectional study was conducted using multi-stage sampling followed by simple random sampling technique. The study was conducted in Aneded woreda, Northwest Ethiopia. A total of 844 reproductive age women were included in the study. Pre-tested semi-structured questionnaire was used to collect the data. Data was collected through face-to-face interviews by 12 data collectors. Data was cleaned, coded and entered into Epi-data, then exported and analyzed using SPSS software. Bivariate and multivariable logistic regression analysis were computed to identify factors related to knowledge of obstetric causes of maternal mortality. The crude and adjusted odds ratios together with their corresponding 95% confidence intervals (CI) were computed. A P-value less than 0.05 was used to declare statistical significance.

**Results:**

This study found that almost half (49.6%) of respondents have good knowledge level towards obstetric causes of maternal mortality. Significant variables associated with knowledge towards obstetric causes of maternal mortality were; being government employee (AOR=3.6, 95% CI=1.4-8.9), respondents who had additional monthly income from family members (AOR=1.54, 95% CI=1.04-2.27), respondents who attended primary school and above (AOR=1.6, 95% CI=1.13-2.25), distance of health facility in which the time it took less than 20 minutes (AOR=2.25, 95% CI(1.24-4.09), 20-39minutes (AOR=3.06, 95% CI=1.66-5.64), 40-60 minutes (AOR=2.38, 95% CI=1.52-5.26), and previous history of prolonged labor (AOR=1.4, 95% CI=1.04 -2.03) were the significant variables.

**Conclusion:**

This study indicated that the reproductive age women in the study area had poor knowledge towards about obstetric causes of maternal mortality. Therefore, to improve maternal knowledge and thereby reduce maternal death, the identified significant factors should be addressed through maternal and child health services. Designing appropriate strategies including the provision of targeted information, education, and communication is important.

## Introduction

World Health Organization (WHO) estimates that 300 million women in developing countries suffer from short and long-term illnesses due to pregnancy and childbirth-related complications. Childbirth is the time of greatest lifetime risk of mortality for the mother and her baby [1]. Since the start of safe motherhood in 1987, countries have made good progress to improve the maternal health and reduce maternal morbidity and mortality. Even if the global rate of maternal mortality ratio has dropped from time to time, the rate of decline is still slow and not satisfactory especially in developing countries, like the Sub-Saharan Africa [2, 3]. Maternal mortality due to direct and indirect obstetric causes is one of the main factors which results in low life expectancy for women. Most of the maternal deaths occur within 24 hours of childbirth, followed by during pregnancy, within seven days of delivery and from two to six weeks after childbirth each accounting for 50%, 25%, 20% and 5% of maternal mortality respectively [4]. In Ethiopia, 20,000 women die each year from pregnancy and childbirth complications with much more maternal morbidity [5]. Among the different strategies which can increase the use of skilled health professionals during pregnancy, labor and delivery and the post-partum period is improving the knowledge level of reproductive age women towards the obstetric danger signs [6–8]. Women and their families should have knowledge of obstetric mortality during pregnancy, delivery and the postpartum period because of the fact that every pregnancy faces risks [9–14]. A community-based study conducted on knowledge about obstetric danger signs among mothers in Tsegedie district showed that the most commonly mentioned danger sign during pregnancy and childbirth was vaginal bleeding. In addition, the study also found that 35.1% and 31.8% of respondents didn't know any danger signs of pregnancy and childbirth [15]. Awareness of obstetric causes of maternal mortality in women of reproductive age is a bottleneck to reduce maternal mortality and to achieve sustainable development goals. Therefore, this study was conducted to assess the knowledge level of reproductive age women and associated factors in Aneded woreda, Northwest Ethiopia.

## Methods

**Study design and area**: A community-based cross-sectional study design was conducted. The study was conducted in Aneded woreda which is found 283 km north of Bahirdar and 282 Northwest of Addis Ababa. The woreda has 20 kebeles. According to population projection of the 2007 national census conducted by the Central Statistical Agency of Ethiopia (CSA), there was an estimated population of 101,734 of whom 50,664 are men and 51,070 were women. From this, 20,693 were reproductive age women. There were four health centers and 20 health posts in the woreda during the data collection period.

**Study period and population**: The study was conducted from 21 February to March 18, 2014. The study population was reproductive age women who were living in the randomly selected kebeles in the woreda. Women who were critically ill, unable to talk or listen were excluded from the study.

**Sample size determination and sampling procedure** : The required sample size was determined using single population proportion formula. The assumptions considered were; proportion (p) of 50%, a margin error of 5%, a design effect of 2 and none response rate of 10%. Accordingly, the sample size was: n = (1.96)²x0.5 (1-0.5) (0.05)² n = 384, And by considering 10% non-response rate and a design effect of 2 the total sample size was 844. A multistage sampling technique was used. First, all the Kebeles in the district were stratified into urban and rural. Then one urban kebele and 5 out of 19 rural kebeles were randomly selected by simple random sampling. List of reproductive age women was extracted from a community-based intervention for action (CBIA) data in the selected kebeles which were collected by health extension workers. The calculated sample size was proportionally allocated to urban (n=75) and rural (n=769) areas respectively.

**Variables of the study**: The dependent variable of this study was knowledge of obstetric causes of maternal mortality. The independent variables were: socio-demographic factors (like; age of mother, religion, residence, marital status, occupation, level of education, monthly income); obstetric history (gravidity and parity), health- system factors (distance, transportation, transport cost, availability health facilities and health professionals), previous self-exposure to hemorrhage, abortion, sepsis, hypertensive disorder of pregnancy and obstructed labor.

**Data processing and analysis**: The collected data was cleaned, coded and entered into EpiData software version 3.1. Prior to the analysis, the whole data were cleaned. The completeness of the data was checked. Errors related to inconsistency were verified using cross tabulation and other data exploration methods. The data was exported to Statistical Package for Social Sciences (SPSS) version 20 software. Then it was recoded, categorized and sorted for further analysis. Descriptive analysis was used to describe the percentages and number distributions of the respondents by socio-demographic characteristics and other relevant variables in the study. Furthermore, logistic regression, specifically Bivariate and multivariable logistics regression analysis were computed to identify factors related to knowledge about obstetric causes of maternal mortality. In bivariate analysis, all variables were entered to the model and variables which had p-value <0.25 were used in multivariable logistic regression analysis. The crude and adjusted odds ratios together with their corresponding 95% confidence intervals were computed. A P-value less than 0.05 was considered to declare a result as a statistically significant variable.

**Ethical consideration**: Ethical clearance was obtained from the Institutional review board (IRB) of Debre Markos University (DMU), College of Medicine and Health science (CMHS). Then officials at different levels in the study area were communicated through letters from DMU, CMHS. Letter of permission was obtained from Aneded woreda health office. Data was collected after informed consent was obtained from each respondent prior to the interview after explaining the purpose of the study, the procedure of data collection, benefits and risks, and confidentiality issues. For respondents who were below 18 years old, consent was obtained from parents/guardians on their behalf. Confidentiality of the information was assured and privacy of the respondents was maintained.

## Results

**Socio-demographic characters**: The respondent rate was 92% out of 844 women. The mean ages of the respondents were 28.93 years (±6.6 SD). Most of the respondents, 643(82.9%) were married and 759(92.9%) were orthodox in religion. More than half participants, 642 (59.5%) couldn't read and write, while 38(4.9%) attended college diploma and above. The majority, 617(79.5%) of the respondents were farmers Table 1.

**Table 1:** socio-demographic characteristics of reproductive age women in Aneded Woreda, Northwest Ethiopia, 2014

Socio-demographic characteristics	Number	Percent
**Age in years**		
15-27	352	45.4
28-38	339	43.7
39-49	85	11.0
**Marital status**		
Married	643	82.9
Single	67	8.6
Widowed	16	2.1
Divorced	47	6.1
Separated	3	0.4
**Religion**		
Orthodox	759	97.8
Protestant	6	o.8
Muslim	11	1.4
**Residence**		
Urban	74	9.5
Rural	702	90.5
**Educational level**		
Can’t read and write	462	59.5
Can read and write	168	21.6
Attend primary school	68	8.8
Attend secondary school	40	5.2
college diploma and above	38	4.9
**Occupation**		
Government Worker	52	6.7
Private Worker	34	4.4
Farmer	617	79.5
Daily Labor	33	4.3
Merchant	40	5.2
**Monthly income (in Ethiopian birr)**		
0-250	65	8.4
251-650	147	18.9
651-1400	376	48.5
1401-2500	161	20.7
2500-10,000	27	3.5
**Additional monthly income from family members**		
Yes	179	23.1
No	597	76.9
**Primary education and above from family members**		
Yes	358	46.1
No	418	53.9
**Radio possession**		
Yes	342	44.1
No	434	55.9
**Television possession**		
Yes	50	6.4
No	726	93.6

**Participants history of exposure to obstetric problems**: Around 631(81.3%) participants had a prior history of pregnancy. Out of this, 156(20.1%) participants had an earlier history of vaginal bleeding during their pregnancy, delivery and/or post-partum period. Only 30(13.3%) had gone to health facility though a large number of participants experienced vaginal bleeding. Out of 631 women who were pregnant, 241(31.1%), 93(12%), 38(4.9%), 28(3.6%) of the respondents had prior history of prolonged/obstructed labor, abortion, hypertension and sepsis, of which only 143(18.4%), 37(4.8%), 26(3.4%), 20(2.6%) had gone to a health facility respectively.

**Knowledge towards obstetric maternal mortality**: More than half, 602(77.6%) knew about obstetric causes of maternal mortality, of which majority 524(67.5%) mentioned obstetric hemorrhage as the main cause. More than half, 482(62.1%) of participants also mentioned prolonged/obstructed labor as the obstetric cause of maternal death, 359(46.3%) as pregnancy-induced hypertension, 361(46.5) as abortion and 250(32.2%) as sepsis Table 2. There were eleven questions related with knowledge towards obstetric causes of maternal mortality and the mean value was 5.3. The minimum knowledge score was 0 and the maximum knowledge score was 11. From the total knowledge score, respondents who answered 5.3-11 questions were categorized as to have good knowledge. Respondents who scored 0-5.3 were grouped as having insufficient knowledge. Accordingly, 383(49.6%) of respondents had a good level of knowledge and 393(50.4%) had insufficient knowledge.

**Table 2:** knowledge of obstetric causes of maternal mortality among Reproductive age women in Aneded District, Northwest Ethiopia, 2014

Knowledge towards obstetric causes	Frequency	percent
**Knew that maternal death can occur due to obstetric causes**		
Yes	602	77.6
No	174	22.4
**Maternal death due to bleeding**		
Yes	524	67.5
No	252	32.5
**Maternal death due to obstructed /prolonged labor**		
Yes	482	62.1
No	294	37.8
**Maternal death due to pregnancy induced hypertension.**		
Yes	359	46.3
No	417	53.7
**Maternal death due to abortion**		
Yes	361	46.5
No	415	53.5
**Maternal death due to sepsis**		
Yes	250	32.2
No	526	67.8
**Maternal death due to sepsis from their family/others they knew.**		
Yes	58	7.5
No	718	92.5

**Factors associated with knowledge towards obstetric causes of maternal mortality**: Variables found to be associated with the dependent variable on bivariate analysis were; age, educational status, occupation type, additional source of family income, family educational status, source of information (radio), distance to health facility, previous contact with health facility, respondents who knew the importance of medical follow-up during antenatal care, delivery and postnatal care, and previous exposure of obstetric complications like; hypertension, prolonged labor Table 3. Significant variables during bivariate analysis were fitted into multivariable analysis. As presented in table 3, the significant variables were; government workers, respondents who had additional monthly income, the presence of family members who attend primary school and above, a short distance of health facility from respondents? residence, and previous history of prolonged labor.

**Table 3:** : logistic regression result for knowledge of direct obstetric causes of maternal mortality and associated factors among Reproductive age women in Aneded District, Northwest Ethiopia, 2014

Variables	Insufficient knowledge	Good knowledge	COR(CI)			
**Occupation**				**P-value**	**AOR(CI)**	**P-value**
Government Worker	37(71.2%)	15(28.8%)	3.337(1.401-7.947)	.003	3.6*9(1.4-8.9)*	0.006*
Private Worker	22(64.7%)	12(35.3%)	2.480(.967-6.364)	.006	*2.4(0.9-6.3)*	*0.075*
Farmer	295(47.8%)	322(52.2%)	1.239(.649-2.366)	.059	*1.2(0.64-2.5)*	*0.486*
Daily Labor	12(36.4%)	21(63.6%)	1.773(.300-1.992)	.515	*0.97(0.36-2.6)*	*0.97*
Merchant	17(42.5%)	23(57.5%)				
**Additional monthly income from Family members**		*1*				
Yes	117(65.4%)	62(34.6%)	2.348(1.659-3.324)		1.54(1.04-2.2)	
No	266(44.6%)	331(55.4%)	1		1	
**primary Education and above from family member**	.000		0.031*			
Yes	211(58.9%)	147(41.1%)	2.053(1.541-2.735)		1.7(1.2-2.3)	
No	172(41.1%)	246(58.9%)	1		1	
**Radio possession**	.000		0.001*			
Yes	189(55.3%)	153(44.7%)	1.528(1.149-2.032)		1.072(.775-1.484)	
No	194(44.7%)	240(55.3%)	1		1	
**Distance of health facility from home (in minutes)**	.004		*.675*			
<20	146(49.0%)	152(51.0%)	2.096(1.205-3.645)	.009	2.254(1.242-4.093)	.008*
20-39	122(54.0%)	104(46.0%)	2.559(1.450-4.518)	.001	3.056(1.656-5.637)	.000*
40-60	93(51.1%)	89(48.9%)	2.280(1.274-4.081)	.006	2.831(1.522-5.266)	.001*
>60	22(31.4%)	48(68.6%)	1		1	
**Importance of following health facility during pregnancy, delivery and postnatal**						
Yes	366(51.1%)	350(48.9%)	2.645(1.480-4.726)		*1.82(0.99-3.3)*	*0.05*
No	17(28.3%)	43(71.7%)	1		*1*	
**History of prolonged labor**	.001					
Yes	140(58.1%)	101(41.9%	1.666(1.225-2.265)	.001	1.4(1.04-2.03)	0.026*
No	243(45.4%)	292(54.6%)	1		1	
**Go to health facility**						
Yes	85(59.4%)	58(40.6%)	1.647(1.140-2.382)		1.033(.597-1.785)	.908
No	298(47. 1%)	335(52.9%)	1		1	
**History pregnancy induced hypertension**	.008					
Yes	27(71.1%)	11(28.9%)	2.634(1.287-5.388)	.008	2.098(0.99-4.44)	*.0.05*
No	356(48.2%)	382(51.8%)	1		1	

## Discussion

Knowledge of obstetric causes of maternal mortality is the first essential step to decrease the highest level of maternal mortality. The findings of this study have provided insight information on reproductive age women's knowledge regarding obstetric causes of maternal death. This study found that 49.6% of women had a good level of knowledge towards obstetric causes of maternal mortality. This finding is low because every reproductive age women should have a better knowledge about obstetric causes of maternal mortality. The finding of this study is lower than a study conducted in Nigeria (69.1%) [16]. This difference could be due to a difference in socio-demographic, socio-cultural and health system factors. Three hundred ninety-three (50.6%) of respondents who lived in the urban area had good knowledge, while 49.3% of rural women had a good level of knowledge towards obstetric mortality. A higher level of knowledge towards obstetric mortality among urban women was also observed in a study done in urban areas of southern Nigeria, 90% [16] and Urban Slum Area of South India, 95.5% [17]. These studies showed a relatively higher prevalence than the current study. This difference could be due to a difference in access to information and health seeking behavior of the community. A similar finding was also observed in a study conducted in Aleta Wondo district, southern Ethiopia showing that pregnant women who reside in urban areas have a higher level of knowledge towards obstetric danger signs than their counterparts in the rural areas [13]. Most of the respondents, (77.6%) had knowledge that maternal death occurs due to obstetric causes. This finding is lower than the study done in India (95.5%) and the study conducted in the delta of Nigeria (96.3%) [16, 17]. The difference could be attributed to the difference in the sociodemographic characteristics, access to information and place of residence. Most of the respondents included in this study were from rural area. More than two third, 67.5% of the respondents mentioned that maternal mortality occurred due to vaginal bleeding. This finding is lower than a study done in the delta of Nigeria (85.4%) [16], and the study conducted in Varanasi, India (73.81%) [18]. The possible reason for the difference could be due to the difference in the sociodemographic characteristics between the study. This finding is higher than a study done in Aletawondo district, Ethiopia (45.9%) [13], Burkina Faso (39.4%) and Guatemala (31%) [19, 20]. The higher level of knowledge in this study compared to the study in Aletawondo could be due to the role of health extension workers, and the community mobilization programs.

More than half of the respondents, 46.3% mentioned pregnancy induced hypertension as a cause of maternal death. This finding is higher than the study done in Aletawondo, Ethiopia (19.5%) [13]. Additionally, 32.2% mentioned that maternal death occurred due to sepsis which is higher than the study done in the delta of Nigeria (4%) [16]. The main source of information for obstetric causes of maternal mortality was from health facilities for 56.8% of participants, from mass media (18.9%), and 0.9% from printed papers. Respondents who were government workers were 3.6 times more knowledgeable towards obstetric causes of maternal mortality than merchants (AOR=3.6, 95% CI=1.4-8.9), and those who had additional monthly income from their family members were 1.54 times more knowledgeable than those who had not (AOR=1.54, 95% CI=1.04-2.2). A study conducted among pregnant women in public health institutions in Mekele city, Ethiopia also showed that government employees were more knowledgeable towards obstetric danger signs [21]. Respondents who had a family member who attended primary school and above were 1.7 times more knowledge than those who hadn?t (AOR=1.7, 95% CI= 1.2-2.3). This may be because of the information that women obtained from their family members. This finding is comparable with a study conducted in Tsegede district of Tigray region, Ethiopia. The study found that mothers who have a formal education were more likely to have higher knowledge towards obstetric danger signs than their counterparts [15]. Similar findings were also observed in studies conducted in Arba Minch town, Tanzania and Egypt [22–24]. Respondents who reside at the distance of less than 20 minutes to reach health facility were 2.25 times more knowledgeable than those who lived greater than the distance of 60 minutes (AOR= 2.25, 95% CI=1.242-4.05). Respondents who live at a distance of 20-39 minutes to reach health facility were 2.7 times more knowledgeable than respondents living at distance 60 minutes distance (AOR=2.7, 95% CI=1.5-5.07). Similarly, those who live 40-60 minutes to reach health facility were 2.59 times more knowledgeable than those who live at 60 minutes distance (AOR=2.59, 95% CI =1.40-4.7). This could be because respondents who lived near health facilities may get more information and can easily access the health facility more easily than those living in a distant area. Respondents who had a previous history of prolonged labor were 1.4 times more knowledgeable than those who had such history (AOR=1.4, 95% CI =1.04-2.03). Women could get more information from health professionals and from health facility due to their previous exposure to health institutions.

## Conclusion

The knowledge level of women towards obstetric causes of maternal mortality in Aneded woreda was comparatively low. Factors associated with knowledge towards obstetric causes of maternal mortality were; Occupation type, educational status, additional income, a distance of health facility and history of prolonged labor. Information regarding obstetric causes of maternal mortality should be provided to the public through available channels such as print and electronic media, billboards and posters, and opinion/religious leaders and through health education. Promotion messages focusing on obstetric causes of maternal mortality and the preventive mechanisms of maternal mortality should be scaled up into the community. Health professionals should provide strong counseling regarding direct causes of maternal mortality. Further comprehensive research should be done on knowledge towards direct obstetric causes of maternal mortality in different settings.

### What is known about this topic

- Obstetric causes are the main cause of maternal death;
- Knowledge of women towards obstetric maternal death is important in the reduction of maternal death.

### What this study adds

- A Large number of reproductive age women have poor knowledge towards obstetric causes of maternal death;
- Knowledge of women towards obstetric deaths can be influenced by several factors including their occupation, educational status, income, a distance of health facility and their obstetric history.

## Competing interests

The authors declare that they have no competing interests.
